# Despite early antiretroviral therapy effector memory and follicular helper CD4 T cells are major reservoirs in visceral lymphoid tissues of SIV-infected macaques

**DOI:** 10.1038/s41385-019-0221-x

**Published:** 2019-11-13

**Authors:** Henintsoa Rabezanahary, Félicien Moukambi, David Palesch, Julien Clain, Gina Racine, Guadalupe Andreani, Ghita Benmadid-Laktout, Ouafa Zghidi-Abouzid, Calayselvy Soundaramourty, Cécile Tremblay, Guido Silvestri, Jérôme Estaquier

**Affiliations:** 1 Centre de Recherche du CHU de Québec, Université Laval, Québec, QC Canada; 2 Yerkes National Primate Research Center, Emory University, Atlanta, GA USA; 3 INSERM U1124, Université Paris Descartes, Paris, France; 4 Centre de Recherche du Centre Hospitalier de l’Université de Montréal, Montréal, QC Canada

## Abstract

Whereas antiretroviral therapy (ART) suppresses viral replication, ART discontinuation results in viral rebound, indicating the presence of viral reservoirs (VRs) established within lymphoid tissues. Herein, by sorting CD4 T-cell subsets from the spleen, mesenteric and peripheral lymph nodes (LNs) of SIVmac251-infected rhesus macaques (RMs), we demonstrate that effector memory (TEM) and follicular helper (TFH) CD4^+^ T cells harbor the highest frequency of viral DNA and RNA, as well of early R-U5 transcripts in ART-naïve RMs. Furthermore, our results highlight that these two CD4 T cells subsets harbor viral DNA and early R-U5 transcripts in the spleen and mesenteric LNs (but not in peripheral LN) of RMs treated with ART at day 4 post infection suggesting that these two anatomical sites are important for viral persistence. Finally, after ART interruption, we demonstrate the rapid and, compared to peripheral LNs, earlier seeding of SIV in spleen and mesenteric LNs, thereby emphasizing the importance of these two anatomical sites for viral replication dynamics. Altogether our results advance understanding of early viral seeding in which visceral lymphoid tissues are crucial in maintaining TEM and TFH VRs.

## Introduction

To date, the identification of cellular and anatomic reservoirs and their eradication remains a major challenge for an HIV cure.^1^ Our understanding of the effect of current drug regimens on virus burden in lymphoid and other tissues is incomplete. Proviral DNA levels are predictive for viral rebound after treatment interruption.^2^ Thus, persistence of HIV proviral DNA is considered as one of the major impediments to eradicate the virus.^3–10^ HIV proviral DNA persists throughout the lives of HIV-individuals, even when treated with antiretroviral therapy (ART), and seems unaffected by ART intensification.^11–15^ Several groups have shown that the viral reservoir (VR) could be maintained by the proliferation of infected cells^16–20^ in which a large majority of provirus is defective due to extensive deletion or hypermutation.^21–24^ Other groups have proposed that ongoing viral replication contributes to the maintenance of the VR,^25–27^ but this has been challenged by others.^28–31^ While the VR is seeded rapidly after infection,^32^ the contributing role of peripheral blood and lymph nodes (LNs) has been challenged by the observation that, in animal models, viral rebound after ART interruption (ATi) could occur in the presence as well as in the absence of viral DNA in either compartment.^32,33^ Thus, viral rebound may originate from anatomical sites that are different from peripheral blood and LNs. Accordingly, actual quantitation of viral DNA in these anatomical sites may not be enough to estimate the overall size of the VR in individuals.^34^ Additional potential candidates for anatomic sites that might contribute to the VR in vivo are visceral lymphoid tissues, which include both the spleen and mesenteric LNs. Mesenteric LNs constitute a specialized lymphoid organ, that is essential in the genesis of the intestinal immune response, as well as in draining the gut-associated lymphoid tissue (GALT). Furthermore, mesenteric LNs are essential for oral tolerance.^35,36^ However, very little focus has been given to these regions in respect to elucidating their role for the VR.

Central memory (TCM) and transitional memory (TTM) CD4 T lymphocytes are the main cellular reservoirs in the blood of ART-treated individuals.^37^ These reservoirs are significantly enriched in CCR6^+^ TCM.^38^ It has been also proposed that HIV reservoirs persist in long-lived stem cell memory CD4^+^ T cells^39^ and in CD4 T cells expressing CD32,^40^ although these results are controversial.^41,42^ Consistent with the fact that HIV targets lymphoid organs, follicular helper (TFH) cells, a subset of memory CD4 T cells, which are mainly localized in germinal centers, have been known to be infected by both HIV and simian immunodeficiency virus (SIV).^43–49^ Recently, analyses of viral sequences in the plasma of viremic controllers have indicated that viral sequences are closer to HIV DNA sequences observed in TFH cells from peripheral LNs, than those observed in CD4 T cells derived from peripheral blood.^50^ However, little is known about the presence of SIV-infected TFH in the spleen and mesenteric LNs under ART, particularly after early ART. Thus, a better understanding of the nature and the dynamics of T-cell subsets involved in early infection and establishment of the tissue reservoir is of crucial importance.

In the present study, we analyzed the extent of early viral dissemination in lymphoid tissues, including mesenteric LNs that drain the small and large intestines and spleen, in comparison to peripheral LNs in nontreated and ART-treated rhesus macaques (RMs). Viral DNA and RNA were analyzed, as well the presence of early R-U5 transcripts in sorted CD4 T-cell subsets. Here, we provide evidence that the frequencies of TEM- and TFH-expressing viral DNA and RNA are higher than that observed in the other T subsets. We also highlight the importance of analyzing mesenteric LNs and the spleen, particularly regarding their importance in terms of viral seeding and cellularity. Our results demonstrate that despite early ART administration, TEM and TFH in the spleen and mesenteric LNs represent VRs. This is in contrast to peripheral LNs in which we detected neither viral DNA nor early R-U5 transcripts. This persistence of viral DNA, detected in these anatomical and cellular sites, may contribute to the viral rebound that occurs early after ATi. We also demonstrate the rapid dissemination of SIV in the different tissues analyzed after ATi, detecting not only viral DNA but also viral RNA. Our results may present a novel avenue to therapeutic strategies that aim to target these populations in visceral tissues.

## Results

### Frequencies of cell-associated viral DNA in lymphoid organs of acutely infected RMs

**Fig. 1:**
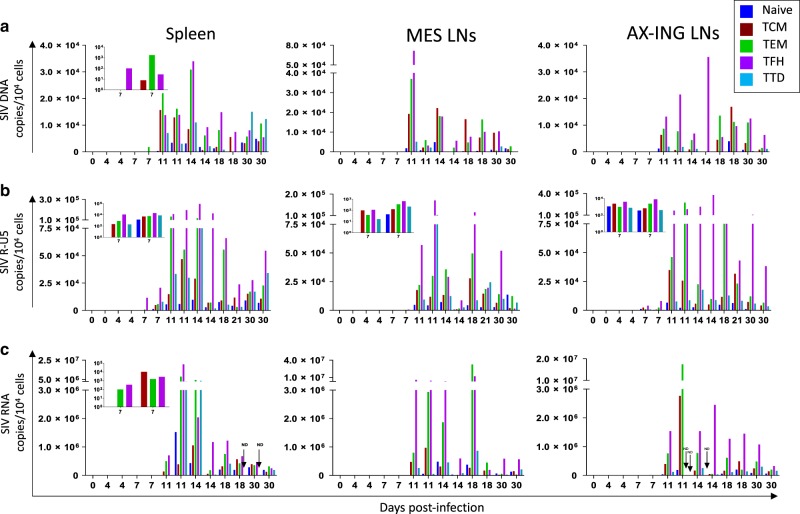
Frequencies of cell-associated SIV DNA, R-U5, and RNA in nontreated SIV-infected RMs. Freshly isolated cells were sorted in Naive, TCM, TEM, TFH, and TTD populations from the spleen, mesenteric LNs (MES LNs) and peripheral LNs (AX-ING LNs) from RMs sacrificed at different times after SIV infection. Each color represents one cell population. **a** Viral DNA was quantified by nested qPCR, and results were expressed as SIV DNA copy numbers per 10^4^ cells. **b** SIV R-U5 was quantified by qPCR, and results were expressed as copies per 10^4^ cells. **c** Viral RNA was quantified by RT-qPCR, and results were expressed as SIV RNA copy numbers per 10^4^ cells

Twelve (12) RMs were infected with 20 AID50 of SIVmac251 and sacrificed at day 4, 7, 11, 14, 18, and 30 post infection, with 2 monkeys examined at each time point to determine the early SIV seeding in lymphoid tissues (Table 1). Viral load (VL) was quantified from sera by quantitative real-time polymerase chain reaction (qRT-PCR) and percentages of CD4^+^CD3^+^ and of CD8^+^CD3^+^ T cells were measured in the blood and lymphoid organs of the different animals by flow cytometry. Five naïve animals were included for comparison. Table 1 summarizes virological and immune parameters. All LNs from different regions were removed, including mesenteric LNs that are disseminated along the colon and in the mesentery and cisterna chyli (Fig. S1).^51^ CD4 T-cell subpopulations from mesenteric and peripheral (axillary and inguinal) LNs and the spleen were sorted using specific antibodies (Table S1). The gating strategy is illustrated in Fig. S2. Subpopulations were defined as: naïve CD45RA^+^CCR7^+^), central memory (TCM, CD45RA^−^CCR7^+^), effector memory (TEM, CD45RA^−^CCR7^−^), terminally differentiated (TTD, CD45RA^+^CCR7^−^) and TFH (CXCR5^+^PD-1^bright^). A qPCR was performed after cell sorting to determine the frequency of SIV-DNA+ T-cell subsets. Viral DNA was detected at day 7, mainly in TEM, TFH, and TCM populations isolated from the spleen of SIV-infected RMs (Fig. 1a); viral DNA was not detected in mesenteric and peripheral LNs at day 7. Thereafter, viral DNA was detected in the spleen, mesenteric and axillary/inguinal LNs (Fig. 1a). The TEM and TFH cell subsets isolated from the spleen and mesenteric LNs in the acute phase showed higher frequencies of viral DNA copies per 10^4^ cells compared to naïve cells, whereas TFH cells of peripheral LNs displayed a higher frequency of SIV-DNA (Fig. S3A, *p* values in red). After 18 days of infection, we observed a reduced frequency of infected cells throughout the various cell subtypes and compartments (Fig. 1a). The TFH population had the highest frequency of SIV-DNA^+^ cells in all the organs studied, whereas in mesenteric and peripheral LNs, TEM and TCM cell subsets also showed a higher frequency of SIV-DNA^+^ compared to naïve cells (Fig. S3B, *p* values in red).

**Table 1:** Virological and immune parameters measured in the blood and lymphoid organs of SIV-infected RMs

Animal OCID	Day of sacrifice	Viral load (copies/ml)	BLOOD				Spleen				MES				AX/ING			
			CD3 CD4 (%)	CD3 CD8 (%)	Ratio CD4/CD8	CD4 (count/mm^3^)	CD3 CD4 (%)	CD3 CD8 (%)	Ratio CD4/CD8	CD4 T-cell numbers	CD3 CD4 (%)	CD3 CD8 (%)	Ratio CD4/CD8	CD4 T cell numbers	CD3 CD4 (%)	CD3 CD8 (%)	Ratio CD4/CD8	CD4 T cell numbers
PB057	0	0.00E + 00	72.1	27.9	2.6	2475	58.5	41.5	1.4	3.36E + 08	59.7	40.3	1.5	7.80E + 08	60.9	39.1	1.6	1.99E + 08
PB061	0	0.00E + 00	72.0	28.0	2.6	2684	55.7	44.3	1.3	3.73E + 08	62.3	37.7	1.6	5.13E + 08	60.6	39.4	1.5	2.07E + 08
PB069	0	0.00E + 00	70.9	29.1	2.4	2191	61.5	38.5	1.6	3.36E + 08	73.4	26.6	2.8	5.68E + 08	71.6	28.4	2.5	8.21E + 07
93750	0	0.00E + 00	55.8	44.2	1.3	1418	66.8	33.2	2.0	3.36E + 08	75.0	25.0	3.0	8.10E + 08	61.1	38.9	1.6	4.33E + 07
528	0	0.00E + 00	49.9	50.1	1.0	1895	62.3	37.7	1.7	3.56E + 08	68.4	31.6	2.2	5.00E + 08	67.3	32.8	2.1	6.39E + 07
NO ART	Days PI																	
PB007	4	0.00E + 00	71.0	29.0	2.4	ND	ND	ND	ND	ND	62.6	37.4	1.7	6.10E + 08	ND	ND	ND	ND
PB038	4	0.00E + 00	62.3	37.7	1.7	ND	62.2	37.8	1.6	1.36E + 08	69.2	30.8	2.2	8.99E + 08	67.2	32.8	2.1	1.51E + 08
PB052	7	7.90E + 04	55.0	45.0	1.2	1382	56.1	43.9	1.3	1.64E + 08	62.9	37.1	1.7	3.14E + 08	63.6	36.4	1.8	1.33E + 08
PB036	7	5.02E + 06	64.7	35.3	1.8	1109	34.9	65.1	0.5	1.17E + 08	72.7	27.3	2.7	6.66E + 08	75.2	24.8	3.0	2.07E + 08
PB041	11	8.94E + 06	65.8	34.2	1.9	555	32.0	68.0	0.5	1.80E + 08	57.6	42.4	1.4	4.27E + 08	55.9	44.1	1.3	3.28E + 08
PB006	11	2.81E + 07	61.5	38.5	1.6	1044	30.5	69.5	0.4	1.11E + 08	54.8	45.2	1.2	3.22E + 08	54.4	45.6	1.2	3.74E + 07
PB051	14	4.67E + 07	73.8	26.2	2.8	917	38.8	61.2	0.6	1.60E + 08	58.5	41.5	1.4	4.07E + 08	73.8	26.2	2.8	1.74E + 08
PB005	14	5.81E + 06	25.0	75.0	0.3	473	49.5	50.5	1.0	3.66E + 08	60.1	39.9	1.5	6.87E + 08	50.3	49.7	1.0	1.28E + 08
PB049	18	9.12E + 07	50.5	49.5	1.0	957	14.8	85.2	0.2	1.83E + 07	54.5	45.5	1.2	3.16E + 08	49.9	50.1	1.0	4.33E + 08
PB021	18	3.70E + 06	38.9	61.1	0.6	494	27.7	72.3	0.4	9.45E + 07	43.3	56.7	0.8	3.62E + 08	44.3	55.7	0.8	3.18E + 08
PB015	30	1.11E + 06	37.0	63.0	0.6	1300	24.1	75.9	0.3	1.37E + 08	47.8	52.2	0.9	3.37E + 08	47.7	52.3	0.9	5.45E + 08
PB033	30	6.54E + 06	29.3	70.7	0.4	673	14.3	85.7	0.2	8.48E + 07	39.0	61.0	0.6	5.34E + 08	39.6	60.4	0.7	1.86E + 08
ART	Days PI																	
R110806	11	5.83E + 02	60.4	39.6	1.5	1393	49.5	50.5	1.0	2.92E + 08	59.0	41.0	1.4	2.47E + 09	59.3	40.7	1.5	1.54E + 07
111466 R	14	0.00E + 00	44.7	55.3	0.8	1550	41.7	58.3	0.7	4.30E + 08	54.5	45.5	1.2	7.20E + 08	52.2	47.8	1.1	1.70E + 08
R110562	27	0.00E + 00	49.4	50.6	1.0	1314	38.6	61.4	0.6	3.60E + 08	55.0	45.0	1.2	4.10E + 08	48.6	51.4	0.9	4.70E + 08
R110360	35	0.00E + 00	54.7	45.3	1.2	734	44.7	55.3	0.8	5.40E + 08	44.9	55.1	0.8	3.60E + 08	61.7	38.3	1.6	5.10E + 08
131660 R	36	0.00E + 00	54.4	45.6	1.2	861	70.0	30.0	2.3	5.39E + 08	69.0	31.0	2.2	8.08E + 08	69.0	31.0	2.2	5.50E + 08
121836 R	55	0.00E + 00	41.0	59.0	0.7	922	45.2	54.8	0.8	3.86E + 08	77.9	22.1	3.5	1.65E + 09	80.7	19.3	4.2	1.35E + 08
ATi	Days post-ART																	
R110482	10	1.10E + 05	59.0	41.0	1.4	2186	50.3	49.7	1.0	4.30E + 08	62.8	37.2	1.7	1.10E + 09	58.1	41.9	1.4	2.10E + 08
121888 R	12	7.66E + 05	59.0	41.0	1.5	1450	47.3	52.7	0.9	9.20E + 08	66.8	33.2	2.0	9.40E + 08	59.7	40.3	1.5	6.55E + 08
R110804	15	3.71E + 03	47.6	52.4	0.9	899	42.9	57.1	0.8	1.50E + 08	60.5	39.5	1.5	7.30E + 08	57.4	42.6	1.3	1.70E + 08
131134 R	15	3.49E + 07	70.3	29.7	2.4	1010	51.0	49.0	1.0	6.96E + 08	76.8	23.2	3.3	8.88E + 08	69.5	30.5	2.3	4.37E + 08
111430 R	18	3.08E + 05	60.5	39.5	1.5	1274	47.7	52.3	0.9	5.40E + 08	59.8	40.3	1.5	1.40E + 09	57.4	42.7	1.3	4.90E + 08

In this table, the time of sacrifice, the viral load in sera, the percentages of CD4 and CD8 T cells among CD3^+^ T cells, the ratio of CD4/CD8, and the numbers of CD4 T cells are indicated from peripheral blood, spleen, mesenteric LNs (MES) and axillary-inguinal (AX/ING) LNs. ART interruption, ATi

HIV reverse transcription is slowed down in quiescent cells, thereby preventing the formation of a full-length double stranded DNA species^4,52,53^ and consequently taking around 3–5 days for completion.^54,55^ Thus, in vitro infection of resting CD4 T cells results in the production of early reverse transcripts that can be marker of early infection.^54–56^ We found that these transcripts are detected in all the T-cell subpopulations starting at day 7 (Fig. 1b). Our results demonstrated that in samples derived from mesenteric and peripheral LNs, in which no viral DNA was detected at day 7 (Fig. 1a), 10^2^–10^4^ copies of R-U5 transcripts (Fig. 1b) can be observed indicating a recent infection. The TFH cells contained the highest levels of these reverse transcripts, over time, compared to naive cells (Fig. S3A, B, *p* values in blue) and their levels are three- to tenfold higher than the level of SIV DNA detected in the same population (Fig. S3A, B). Furthermore, contrasting with the dynamics of cell-associated SIV DNA, we did not observe a major reduction in the levels of early reverse transcripts after the peak of viral replication, suggesting that a large proportion of CD4 T cells continue to be infected (Fig. 1b and Fig. S3B). Altogether, our results demonstrate the early establishment of cell-associated SIV DNA in different anatomical lymphoid sites targeting TEM, TCM, and TFH cell populations.

### Frequencies of cell-associated viral RNA in lymphoid organs of acutely infected RMs

We then assessed viral RNA in the different CD4 T-cell subsets. Cell-associated RNA was detected from day 7 onward within splenic TEM, TCM, and TFH cell subsets (Fig. 1c). From days 11–14, the TFH cell population was the subset expressing the highest level of viral RNA in the spleen, but also in peripheral LNs and in mesenteric LNs compared to naive cell subset (Fig. S4A). At the initial steady state phase of infection, a drastic reduction of viral RNA was observed in TEM and TFH cells, both in the spleen in peripheral LNs and in mesenteric LNs. TFH cells express the highest levels of SIV RNA among all T-cell subsets examined (Fig. S4B). Interestingly, one animal (#PB049) displayed high levels of cell-associated viral RNA in mesenteric LNs (both TEM and TFH populations) at day 18, which coincided with a high VL (9.10^7^ copies/ml) (Fig. 1c). In situ hybridization of the spleen and mesenteric LNs in acutely infected RM (Fig. S5) showed the presence of SIV-RNA+ cells in the follicle as individual spots (Fig. S5), and not a diffuse signal corresponding to the accumulation of viral particles trapped in the follicle, is consistent with a previous report,^57^ and with the detection of viral RNA in TFH cells. Taken together, our results demonstrate that TFH and TEM cell subsets are the most prominent T cells expressing viral RNA in lymphoid tissues early after infection.

To evaluate the capacity of TCM, TEM, and TFH cells to produce infectious virus, cell subsets isolated from mesenteric LNs of SIV-infected RMs were sorted and activated using plate-bound anti-CD3 and anti-CD28 antibodies. Viral DNA content was evaluated by qPCR before stimulation, showing similar levels in the three cellular subpopulations (Fig. S6A). Upon 4 days of stimulation, viral production was measured by qRT-PCR in the culture fluids. The three subpopulations produced high levels of SIV, but statistically higher levels were obtained from TFH cells (Fig. S6B). To evaluate the infectious capacity of virus produced by the different subpopulations, culture supernatants were used to infect CEM×174 cells and cell-associated viral DNA was quantified after 4 days. We observed that all three subpopulations produced viruses, which were infectious for the reporter cell line (Fig. S6C). These results demonstrate that TFH and TEM are preferential SIV targets early after infection in visceral lymphoid tissues and that integrated proviruses in these cells are fully competent for RNA production and capable of releasing infectious viral particles.

### Pool of SIV-infected CD4 T-cell subpopulations in RMs

**Fig. 2:**
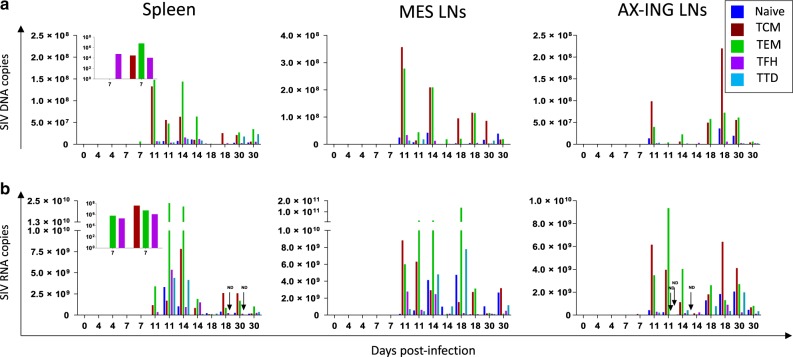
Total amount of SIV DNA and RNA in nontreated SIV-infected RMs. Cells as described in Fig. 1 were sorted and each color represents one cell population. **a** Total amount of SIV DNA copies in the different tissues. **b** Total amount of SIV RNA copies in the different tissues. ND not done due to the low number of cells recovered

After SIV infection, spleen and LNs are enlarged^58^ and associated with increased tissue fibrosis^59^ whereas effector memory cells, including TEM and TFH, are depleted early after SIV infection,^46,51,60–62^ this makes it necessary to determine the absolute numbers of T lymphocytes after cell isolation. We then determined the absolute numbers of each T-cell subset within the different tissues analyzed. Thus, all the LNs localized in the axillary and inguinal regions and mesenteric LNs, including the cisterna chyli, were recovered upon euthanasia. Totally, 30–40 LNs were recovered from the mesenteric region per SIV-infected RM, compared to only 20 LNs in healthy RMs. This LN expansion was also associated with enlarged cisterna chyli. Thus, several billions of cells were recovered from the mesenteric region. The submandibular and mediastinal LNs were not analyzed in these monkeys. In Table 1, as well in Fig. S7, we reported the total numbers of CD4, TCM, TEM, and TFH cell subsets recovered from each compartment. Our results indicate that total CD4 T-cell counts as well TEM and TFH declined at the initial steady state of infection in the spleen, whereas a slight increase in the number of CD4 T cells derived from axillary and inguinal LNs compared to healthy individuals was observed (Fig. S7). In mesenteric LNs, we also detected a reduction in TEM and TFH cells. Based on T-cell numbers and the frequency of cell-associated SIV DNA and RNA within each population, we then calculated the pool of SIV DNA and RNA cells for each RM (Fig. 2a, b). TCM and TEM cells represent the main populations expressing SIV-DNA in all the tissues analyzed both during acute and steady state phases of infection (Fig. 2a, whereas TEM cells represent the most abundant SIV-RNA population in lymphoid tissues (Fig. 2b).

**Fig. 3:**
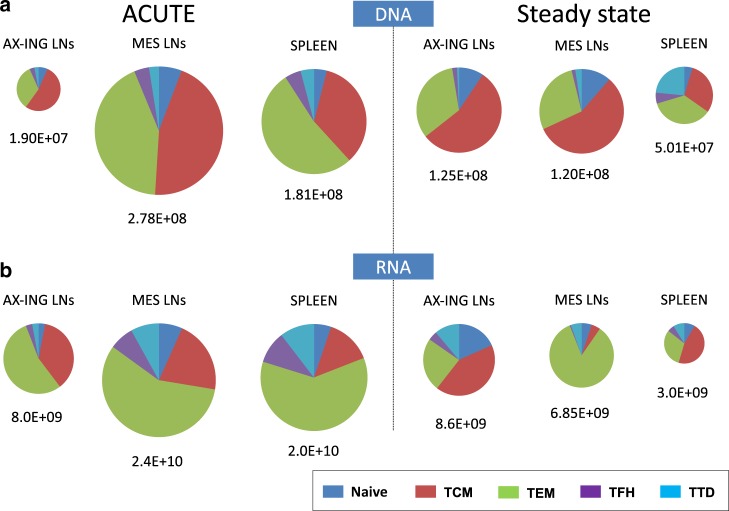
Distribution of SIV DNA and RNA in cellular and anatomical sites. The pie graphs represent the distribution of SIV **a** DNA and **b** RNA per subpopulation per organ for the acute (days 11–14) and steady state (days 18–30) phases of infection. Each graph represents the median of four animals. The size is proportional to the total amount of SIV DNA or RNA in total CD4 T cells per organ (the absolute value is indicated below each graph and the size of the Ax/Ing LNs pie graph at the acute phase is used as reference)

We also calculated the total burden of SIV DNA and RNA per organ during either the acute (days 11–14) or initial steady state (days 18–30) phases of infection (Fig. 3a). We found that mesenteric LNs and spleen represented the main anatomical SIV DNA reservoirs at the peak (days 11–14), accounting for 14.6- and 9.5-fold more DNA than peripheral LNs (*p* = 0.0054). Thereafter, total levels of SIV-DNA within mesenteric LNs decreased by half, compared to the acute phase, and by approximately 75% within the spleen (Fig. 3a). On the contrary, an increase in the total quantity of cell-associated SIV DNA was observed from the acute to the initial steady state phase of infection in peripheral LNs, mostly driven by the increased number of CD4 T cells (Table 1). In all tissues analyzed, TCM and TEM represented approximately 80% of the SIV DNA cellular reservoirs, regardless of the infection stage. Furthermore, our results indicate that the proportions of cells expressing viral RNA in mesenteric LNs and in the spleen were 2.9- and 2.4-fold higher than in peripheral LNs at the peak of infection (Fig. 3b, left panels). However, the most important observation was probably that the pool of cell-associated SIV RNA in the spleen declined drastically by more than 85%, concomitantly with the decline in cell-associated SIV-DNA (Fig. 3b, right panels). This can be explained by the early depletion of CD4 T cells in this anatomical compartment (Fig. S7). In terms of T-cell subsets, TEM was the main SIV RNA+ population during the acute phase. Thus, whereas TEM are almost 40% of viral DNA, 70% of the pool of viral RNA is related to the TEM subset (Fig. 3a, b). At the initial steady state phase of infection, more than 80% of SIV-RNA^+^ cells are TEM in mesenteric LNs, (Fig. 3b). Even though TFH cells have a higher frequency of cell-associated SIV-DNA and a higher capacity to produce virus than TEM cells (Fig. S6), this population was minor, compared to the total numbers of TEM cells. These results shed light on the importance of each lymphoid compartment in contributing to the pool of transcriptionally active reservoirs. Altogether, these results highlight the importance of analyzing individual tissues, particularly the visceral lymphoid tissues, in regard to their relative importance for viral seeding and cellularity early after infection.

### Memory CD4 and TFH represent the main VRs in visceral lymphoid tissues in early ART-treated SIV-infected RMs

Next, we decided to determine the impact of ART therapy on viral seeding. Indeed, a critical barrier to an HIV-1 cure is the establishment of a VR in quiescent CD4 T lymphocytes. First, we determined the presence of viral DNA and R-U5 transcripts in CD4 T cells sorted from peripheral blood (Fig. S8A) and LNs (Fig. S8B) from RMs treated with a cocktail of antiretroviral ART drugs at week 6 post infection (Table 2). We found, as expected, that cell-associated viral DNA was reduced after ART, both in blood at weeks 14 and 32 (week 32, 0.97 × 10^3^ copies/10^4^ cells vs. 10.8 × 10^3^ copies/10^4^ cells at week 6) (Fig. S8A) and in peripheral LNs (week 32, 2.8 × 10^3^ copies/10^4^ cells vs. 18.6 × 10^3^ copies/10^4^ cells at week 6) (Fig. S8B). In comparison, whereas in nontreated RMs the frequencies of early reverse transcripts in the blood and LNs reached 2.5 × 10^4^ copies/10^4^ cells and 3.5 × 10^4^ copies/10^4^ cells, respectively, these levels decreased after ART, but remained elevated even after 32 weeks of therapy (higher than 5 × 10^3^ copies/10^4^ cells). Extending our analyses to the spleen at week 36, the frequencies of viral DNA were quite similar in the different compartments, as well the frequencies of the reverse transcripts (Fig. S8C). Thus, SIV is seeded in CD4 T cells from all the tissues analyzed in RMs treated with ART at week 6 post infection.

**Table 2:** Viral load CD4 and CD4 T cell counts in SIV-infected RMs treated with ART at week 6

Animal	Weeks post infection	Viral load copies/ml	CD4 count
RRe15	6	3,250,000	484
	14	209	331
	32	30	1014
RBo15	6	2,620,000	133
	14	30	408
	32	30	591
RCy15	6	245,000	337
	14	30	524
	32	30	1332
REb16	6	2,440,000	723
	14	166	1122
	32	30	1473
RHa15	6	246,000	262
	14	30	591
	32	30	740
RHb15	6	577,000	560
	14	30	845
	32	30	660
RHo15	6	1,600,000	253
	14	182	708
	32	30	559
	36	30	815
RJl15	6	1,960,000	375
	14	276	574
	32	30	469
	36	30	266
RAk15	6	199,000	726
	14	30	1111
	32	30	1015
	36	30	651
RNi15	6	309,000	424
	14	78	609
	32	30	287
	36	30	625
RWl15	6	442,000	986
	14	63	1250
	32	30	1547
RYj15	6	128,000	778
	14	30	1199
	32	30	726

RMs (*n* = 12) were followed still week 32, and four animals are sacrificed at week 36. The viral load in the blood and CD4 T cell counts are indicated at each time point post infection

**Fig. 4:**
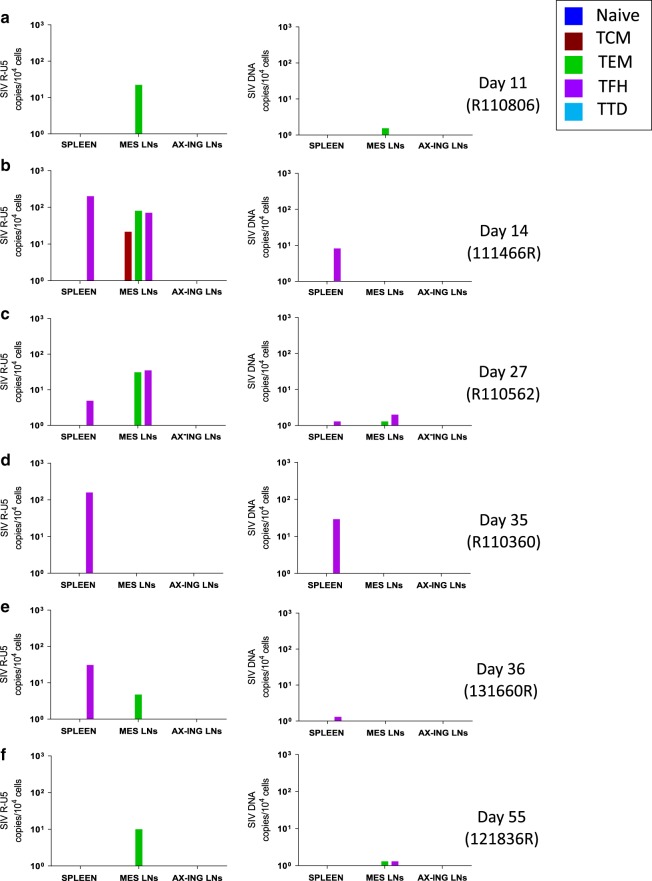
Detection of SIV DNA and R-U5 in RMs treated with ART at day 4 post infection. RMs were treated with ART at day 4 post infection. ART consists in a cocktail of TFV and FTC administrated subcutaneously, and of RGV and RTV administrated by oral route. IDV was administrated during week one. ART was administrated once per day. Cells were isolated from the spleen, the MES and AX-ING LNs of six ART-treated RMs. RMs were sacrificed at different time points, post infection. After cell sorting, early reverse transcripts and total SIV DNA levels were quantified by qPCR in Naive, TCM, TEM, TFH, and TTD cells. SIV R-U5 and DNA results are expressed as copies per 10^4^ cells. ND not done due to the low number of cells recovered. **a** #R110806, **b** #111466R, **c** #R110562, **d** #R110360, **e** #131660R, and **f** #121836R

Next, we assessed the impact of ART administrated early after infection in RMs (such as day 4). Indeed, it was previously shown that early ART did not completely eradicate SIV.^32,33,63^ We observed at the time of sacrifice that viremia was not detected in five RMs out of six (limit of detection 50 RNA copies/ml). In #R110806 sacrificed at day 11 after infection, and treated with only 7 doses of ART, VL was 5.8 × 10^2^ copies/ml, which is at least 3 log lower as compared to nontreated RMs (Table 1). In contrary to ART administrated at week 6 post infection (Fig. S8C), we did not detect any viral DNA in CD4 T cells from peripheral blood (data not shown) from RMs treated with ART at day 4, even in those (#R110806 and #111466R) that received a short course of treatment (respectively 7 and 10 days of ART). This suggests that such early ART combination had a strong inhibitory effect on viral infection. Therefore, we decided to analyze infected CD4 T-cell subsets in the spleen and in mesenteric and peripheral LNs. We clearly identified cell-associated SIV DNA in these two anatomical visceral compartments in the six ART-treated monkeys compared to the peripheral LNs (Fig. 4). The main subsets expressing SIV DNA were TEM and TFH cells (Fig. 4); the latter was found only in the spleen. Thus, whereas in nontreated animals the levels of SIV DNA were around 10^4^ copies/per 10^4^ cells (Fig. 1a and Fig. S3), only less than 10^2^ copies/per 10^4^ cells were detected in ART-treated RMs (Fig. 4). However, given the number of TEM and TFH cells in these organs under ART, and considering one copy of viral DNA per cell, the pool of cell-associated SIV DNA represents a total of 78,300, 8200, and of 21,500 TEM cells in mesenteric LNs of RMs (#R110806, #R110562, and #121836R, respectively), and of 39,900, 700, 110,800, and 28,100 TFH cells in the spleen of RMs (#111466R, #R110562, #R110360, and #131660R, respectively). Our results indicate the absence of detection of SIV DNA from the sorted TCM (CD45RA^−^CCR7^+^) cell population in the six ART-treated RMs. Our results also demonstrate the presence of R-U5 transcripts both in the spleen and mesenteric LNs of ART-treated RMs (Fig. 4). TEM and TFH cells displayed most of these RU-5 transcripts, which may be indicative of recent infection of these CD4 T cells in both compartments. We also assessed whether TFH cells express CD32. However, as shown in Fig. S9, TFH cells are not enriched in CD32 expression compared to the other CD4 T cell subsets. The majority of the CD32^+^CD3^+^CD4^+^ expressed CD20 a marker of B cells that could indicate a membrane transfer namely trogocytosis. However, this population once sorted from mesenteric LNs was negative for SIV-DNA in ART-treated RMs (Fig. S9). Furthermore, SIV DNA was not detected in CD3^+^CD4^−^ sorted populations (data not shown).

**Fig. 5:**
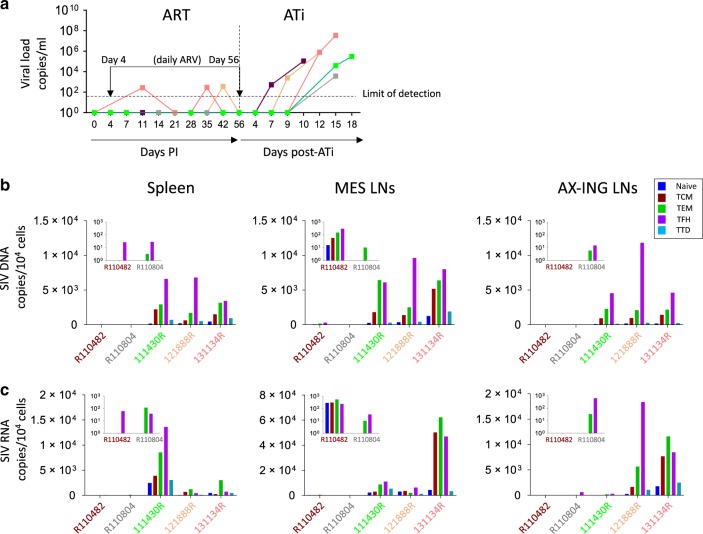
Viral load and frequencies of cell-associated SIV DNA and RNA after ART interruption. RMs were sacrificed at different time points after ATi (#R110482, at day 10; #R110804, at day 15; (#111430R, at day 18; #121888R, at day 12 and #131134R, at day 15). **a** Viral loads during ART and after ATi were quantified by qRT-PCR. Each color represents one individual. Frequencies of **b** SIV DNA and **c** SIV RNA were quantified by qRT-PCR in sorted CD4 T-cell subsets. Each color represents one cell population. RMs are indicated by the same color as in panel (**a**). Results are expressed as copies per 10^4^ cells

Having shown that TEM and TFH cells represent the main VRs in visceral lymphoid tissues in early ART-treated SIV-infected RMs, we decided to interrupt ART in RMs controlling viremia after 8 weeks of ART in order to assess early SIV seeding. RMs were sacrificed at days 10, 12, 15, and 18 post ATi (Table 1). Thus, we observed a viral rebound in two weeks (Fig. 5a). Furthermore, although this study cannot be a longitudinal analysis, our results indicate a distinct RM profile. Thus, in #R110482 sacrificed at day 10, with a VL reaching 1.1 10^5^ copies/ml, we observed viral DNA in splenic TFH only, but more diverse in mesenteric LNs including also naïve CD4 T cells; none of them expressed viral DNA in peripheral LNs (Fig. 5b). In #R110804 sacrificed at day 15, with a VL reaching 3.7 × 10^3^ copies/ml, TEM and TFH cells expressed viral DNA, both in the spleen and mesenteric LNs, but also in peripheral LNs. In the three additional RMs, who displayed higher viremia, we detected viral DNA in all the CD4 T-cell subsets sorted from the spleen, mesenteric and peripheral LNs (Fig. 5b). Furthermore, we also demonstrate that these CD4 T-cells subsets also expressed viral RNA (Fig. 5c) suggesting productively infected cells, and early step in viral rebound in RMs #110482 and #110804. In RMs #131134R, the extent of viral RNA was at least fivefold higher in mesenteric LNs compared to the other tissues analyzed.

Taken together, our results indicate that in early ART-treated SIV-infected RMs, mesenteric LNs and spleen represent potential tissue VRs, and that TFH and TEM constitute the main cellular reservoirs in those tissues. In addition, we demonstrate the rapid seeding of SIV after ART interruption, underlining the importance of these two anatomical sites for viral replication dynamics.

## Discussion

Animal models such as the rhesus macaque offer the opportunity to identify and comprehensively study infected organs that are typically unavailable in human studies. This is of crucial importance, given that recent reports, in both SIV-infected RMs^32,33^ and humans,^64–66^ indicated the absence of complete viral eradication following early treatment initiation. In this study, we report the early seeding of SIV in lymphoid tissues and demonstrate that TFH and TEM feature the highest viral burden of SIV-infected cell populations. Our study identified the spleen and mesenteric LNs, in contrast to peripheral LNs (axillary and inguinal LNs), as lymphoid compartments of viral persistence despite early ART initiation, emphasizing the importance of these visceral lymphoid tissues. In addition to the spleen, mesenteric LNs represent a major site for TFH cells. Mesenteric LNs are located along the intestine and drain the small and the large intestine. They connect to the left subclavian vein through the thoracic lymphatic duct, which is the most common drainage trunk of most of the body’s lymphatics,^67,68^ and therefore they may contribute to viral dissemination. Importantly, our data highlight that viral dissemination is occurring rapidly after ATi in these visceral lymphoid tissues and earlier than in peripheral LNs, showing a diversity of productively infected CD4 T-cell subsets.

A high level of cellular activation in mesenteric LNs has been previously reported using positron emission imaging (PET) scan performed in chronically SIV-infected RMs. In situ hybridization after necropsy has also revealed a high level of viral replication in these lymphoid organs in monkeys.^69,70^ Our results show that at the peak of infection, CD4 T cells in mesenteric LNs represent a large site of viral replication. The notable reduction in the number of splenic CD4 T cells could be related to extensive viral replication-mediated cell death.^71–74^ However, at the steady phase of infection, the pool of SIV-DNA cells was quite similar in mesenteric and peripheral LNs.

Interestingly, in ART-suppressed infant SIV-infected RMs, treated at week 3 after infection,^75^ or here in RMs treated at week 6 post infection, the levels of cell-associated viral DNA were similar in CD4 T cells isolated from the different tissues such as blood, peripheral LNs, and the spleen. In contrast, the administration of early ART, a situation not often occurring in HIV-infected individuals, resulted in limited viral seeding in the spleen and mesenteric LNs as well as in the absence of viral DNA detection in blood and peripheral LNs. Although connected to mesenteric LNs, we did not address the role of Peyer’s patches, which are concentrated in the distal part of the ileum, but they will be of interest for future study. Thus, despite of fully suppressed viral replication as demonstrated by the absence of viral viremia in the sera as well as in peripheral LNs, which is consistent with a previous report,^33^ viral rebound observed after ATi is highly indicative of this persistence.

Our data also elucidated the infected CD4 T cells that are involved in the maintenance of VRs in visceral lymphoid tissues. Our results show that TEM (CD45RA^−^CCR7^−^) and TFH cells are the main infected cell populations. These results may suggest that in lymphoid tissues, TEM and TFH cells could be less sensitive to ART inhibition than other cell subsets or express less restriction factors such as SAM domain and HD domain-containing protein 1 (SAMHD1).^76^ In infant RMs treated with ART, viral RNA was detected in lymphoid and nonlymphoid tissues indicating the persistence of viral replication despite the absence of viral detection in the plasma.^75^ Estes et al.^26^ reported that the detection of SIV-RNA^+^ in ART-treated RMs could be related to a lower levels of ART in the tissues analyzed.^26^ Herein, we detected R-U5 transcripts in CD4 T cells isolated from the mesenteric LNs and the spleen of early ART-treated RMs. Although the detection of R-U5 transcripts may represent dead-end viruses, their detection in TEM and TFH may represent remnants of recent infections despite ART. The persistence of a low level of viral replication in these cellular compartments could be responsible for the rapid rebound of SIV upon discontinuation of effective antiviral therapy.

Other groups have proposed that the VR in blood could be maintained by the proliferation of infected cells^16–20^ in which a large majority of provirus is defective with extensive deletion or hypermutation.^21–24^ The observations that latent infected cells can proliferate in response to cytokine without viral production while retaining the ability to produce virus following subsequent TCR stimulation^21,77^ may support the hypothesis that TFH cells, which are localized in the B-cell follicles, may allow to maintain and to replenish VRs. Indeed, TFH cell are more prone to produce virions than TCM or naïve cells, a difference that could be explained by differences in epigenetic and chromatin states in these populations.^78,79^ The observation that we detected viral RNA earlier in visceral lymphoid tissues than in peripheral LNs shortly after ATi, is consistent with an earlier reactivation of those cells once ART is interrupted in these visceral lymphoid tissues.

Due to limited/restricted dissemination of free viral particles in the tissues, the detection of R-U5 transcripts, possible remnants of recent infection, could be indicative of a viral source closer to such subpopulations. Although, it cannot be excluded that the persistent infection of CD4 T cells could be intrinsic to CD4 T cells in which more than 98% of viral DNA is defective,^21–24^ additional cells could participate in this vicious cycle, feeding new infections. It has been previously shown that macrophages efficiently transmit HIV to T cells.^80^ Tissue-resident macrophages are long-lived cells that are capable of harboring virus,^81^ and recent evidence indicates that they contain lower intracellular concentrations of antiretroviral drugs than those observed in CD4 T cells.^82^ Thus, we cannot exclude the possibility that tissue-resident macrophages may represent a potential residual source of viral dissemination under ART.

This study hereby provides a comprehensive overview about the early dissemination of SIV in which (i) TFH and TEM are the infected cells with the highest levels of both viral DNA and RNA and (ii) visceral lymphoid tissues play a major role on the establishment of VRs. Furthermore, to our knowledge very few reports have actually addressed infected cells after ATi. Thus, the demonstration of the rapid seeding of SIV upon ART interruption, in which TEM and TFH from visceral lymphoid tissues produce viral RNA earlier than peripheral LNs, contribute to the understanding of viral replication dynamics. Therefore, our results may provide a novel avenue to therapeutic strategies that aim to target these CD4 T-cell populations in visceral lymphoid tissues thereby overcoming this obstacle for complete virus eradication.

## Materials and methods

### Animals, viral inoculation, and sample collection

Twenty-eight RMs seronegative for SIV, STLV-1 (Simian T Leukemia Virus type-1), SRV-1 (type D retrovirus), and herpes-B viruses were used in this study including five healthy RMs and 23 RMs infected intravenously with SIVmac251 (20 AID_50_) (Laval University). Eleven of them were treated daily with ART (Table 1). At day 4 post infection, RMs were treated with Tenofovir (TFV, 20 mg/kg, GILEAD) and Emtricitabine (FTC, 40 mg/kg, GILEAD) subcutaneously and Raltegravir (RGV, 20 mg/kg, MERCK) and Ritonavir (RTV, 20 mg/kg; Abbvie) by oral route. Indinavir was administrated only during the week 1 (IDV, 2 mg/kg, MERCK). RMs were euthanized at different time point post infection as shown in Table 1. Lymphoid tissues, including spleen, mesenteric LNs including cisterna chyli and axillary/inguinal LNs were recovered immediately after euthanasia for cellular analysis. Cell numbers were calculated from LNs retrieved in each region (inguinal and axillary LNs, ileum and colon mesenteric LNs; the totality of the LNs were retrieved, Fig. S1). Cells isolated after mechanical process were counted.^83^ Tissues were not digested with collagenase or other proteases to limit negative effects on the expression of cell surface markers. For each blood-sampling point, a hemogram was performed using an Abaxis VetScan HM5 hematology instrument (Abaxis, CA).^46^

In addition, twelve SIV-infected RMs were treated with ART at week 6 post infection (Yerkes National Research Center). ART included 20 mg/kg Tenofovir (TFV), 40 mg/kg Emtricitabine (FTC), and 2.5 mg/kg Dolutegravir (DTG), and was administered once a day via subcutaneous injection with Darunavir (DRV) orally twice daily until necropsy. Peripheral blood mononuclear cells (PBMC) and LNs were recovered for cellular analysis at weeks 6, 14, and 32. At week 36, 4 RMs were sacrificed and the spleen, PBMC, and LNs were recovered (Table 2).

### Ethics statement

RMs were housed at University Laval in accordance with the rules and regulations of the Canadian Council on Animal Care (http://www.ccac.ca). The protocol was approved by the Laval University Animal Protection Committee (Project number 106004). Animals were fed standard monkey chow diet supplemented daily with fruit and vegetables and water ad libitum. Social enrichment was delivered and overseen by veterinary staff and overall animal health was monitored daily. Animals were evaluated clinically and were humanely euthanized using an overdose of barbiturates according to the guidelines of the Veterinary Medical Association.

At Yerkes National Primate Research Center, animal studies were conducted strict accordance with USDA regulations and the recommendations in the *Guide for the Care and Use of Laboratory Animals* of the National Institutes of Health, and it was approved by the Emory University Institutional Animal Care and Use Committee (project YER-2002891-ENTRPR-A). Commercial dry food supplemented with fruit was provided by the veterinary personnel, and water was available ad libitum. SIV-infected RMs are singly caged but have visual, auditory, and olfactory contact with at least one social partner, permitting the expression of noncontact social behavior. Animal cages also accommodate additional animal enrichment, including objects such as perching and other manipulanda. Animal welfare was monitored daily. Appropriate procedures were performed to ensure that potential distress, pain, or discomfort was alleviated. The sedatives ketamine (10 mg/kg) and telazol (4–5 mg/kg) were used for blood draws and biopsy specimens. Euthanasia of RMs, using pentobarbital (100 mg/kg) under anesthesia, was performed only when deemed clinically necessary by veterinary medical staff and according to IACUC endpoint guidelines.

### Cell sorting

Cells derived from either spleen or LNs (10^8^ cells) were sorted using a BD Influx cell sorter (Becton Dickinson) using specific antibodies: anti-CD3, CD8, CD4, CD20, PD-1, CXCR5, CD45RA, and CCR7 mAbs (Table S1). Sorted populations, included TFH cells (CXCR5^+^PD-1^bright^), naive (TN, CD45RA^+^CCR7^+^), central memory (TCM, CD45RA^-^CCR7^+^), effector memory (TEM, CD45RA^−^CCR7^−^) and TTD (CD45RA^+^CCR7^−^) (Fig. S2). CD3+ CD20+ CD32+ cells were sorted in addition to the other CD4 T-cells subsets. Samples were preserved at −80 °C until used for DNA analysis or in trizol (Invitrogen) for RNA quantitation.

### Viral RNA quantification

VLs in the sera of SIV-infected RMs and cell culture supernatants were quantified by RT-qPCR using PureLink Viral RNA/DNA Kit (Invitrogen). The PCR mixture was composed of 4× TaqMan Fast Virus 1-Step Master Mix (Applied Biosystems), 750 nM of primers and 200 nM of probe. Primers and probe sequences are listed in Supplemental Table 2. A plasmid encoding for *gag* gene of SIVmac251 was used as a standard. Serial tenfold dilutions of SIVmac251 plasmid were performed to generate a standard curve, starting at 10^9^ copies/µl of SIV. Amplifications were carried out with a 7500 Real-Time PCR System (Applied Biosystems), using the following parameters: 50 °C/5 min, 95 °C/20 s, and 40 cycles (95 °C/15 s, 60 °C/1 min). Samples were run in duplicates and results are expressed as SIV RNA copies/ml.^84^

SIV cell-associated RNA quantification was performed from 10^5^ sorted cells. Viral RNA was extracted with trizol procedure according to the manufacturer’s instructions and resuspended in 50 µl of RNase-Free water (Invitrogen). DNA traces were eliminated from RNA samples using a Turbo-DNA free kit (Invitrogen). For RNA quantification, 5 µl of RNA were amplified by RT-qPCR. Eukaryotic 18S rRNA Endogenous Control mix (Applied Biosystems) was used for estimating cell number in each sample. Samples were run in duplicates and results are expressed as number of SIV RNA copies per 10^4^ cells.

### Viral DNA quantification

For SIV DNA quantification, DNA was purified from frozen pellets of sorted cell subpopulations (10^5^ cells from untreated RMs and 5 × 10^5^ to 10^6^ cells from ART-treated RMs) using the Genomic DNA Tissue kit (Macherey Nagel). DNA was eluted with 50 µl of buffer elution and 10 µl of SIV DNA was amplified by nested PCR with SIV251-specific primers surrounding the *nef* coding region.^83^ A first round of PCR was performed using 50 nM of preco and K3 primers, 10× PCR buffer, 2 mM MgCl_2_, 1.25 U of AmpliTaq (Applied Biosystems), 0.8 mM DNTP (Invitrogen) in a Biometra thermocycler using the following parameters: 95 °C/1 min 45 s, 45 cycles (95 °C/30 s at, 60 °C/30 s and 72 °C/1 min 10 s), 72 °C/6 min. Totally, 5 µl of PCR product were re-amplified using 250 nM of SIV-DNA primers and probe and 2× PrimeTime^®^ Gene Expression Master Mix (IDT) in a 7500 Real-Time PCR System (Applied Biosystems). PCR amplification parameters were 95 °C/3 min, 45 cycles (95 °C/15 s, 60 °C/1 min). Samples were run in duplicates except samples from ART-treated RMs, which were run in quadruplicate due to the low levels of viral DNA. For DNA quantitation, serial dilutions of a plasmid were performed to generate a standard curve ins.

To estimate cell number in each sample and as an internal control, ribosomal 18S DNA was amplified in parallel, with 2× Quantitec Sybr Green PCR Kit (Qiagen) and 250 nM of specific primers in a 7500 Real-Time PCR System (Applied Biosystems). Termocycling settings consisted of 50 °C/2 min, followed by 95 °C/15 min, 40 cycles (94 °C/15 s at, 60 °C/30 s and 72 °C/35 s), followed by a dissociation stage. A standard curve was used to estimate cell numbers and the results were expressed as SIV DNA copies per 10^4^ cells. Primers and probe sequences are listed in the Table S2.

### SIV RU-5 transcripts quantification

Cell pellets from ART-naive SIV-infected RMs were resuspended in 50 µl lysis buffer (10 mM Tris [pH 8.0]; 0.5 mM EDTA; 0.0001% SDS; 0.001% Triton; 100 µg/ml Proteinase K), incubated 3 h at 50 °C and 10 min at 95 °C.^85^ SIV R-U5 and 18 S DNA were quantified in parallel using 5 µl of samples amplified in duplicate using 2× Quantitec Sybr Green PCR Kit (Qiagen) and 250 nM of specific primers in a 7500 Real-Time PCR System (Applied Biosystems). The following parameters were used: 50 °C/2 min, followed by 95 °C/15 min, 40 cycles (94 °C/15 s at, 60 °C/30 s and 72 °C/35 s), followed by a dissociation stage. A plasmid, containing the early reverse transcripts, was used to generate a standard curve. Results are expressed as SIV R-U5 copies/10^4^ cells. Primers sequences are listed in the Supplemental Table 2. Because these primers can also detect viral DNA due to the 2 LTR, a ratio higher than 3 was considered significant for comparing R-U5 transcripts and viral DNA.

Furthermore, for ART-treated RMs, the samples are the same as described for DNA, purified with the DNA Genome Kit (Macherey Nagel) and R-U5 transcripts were quantified in comparison to viral DNA. In order to increase the probability to detect SIV under treatment, the number of cells in each aliquot were increased (10^5^ cells from untreated RMs and 5 × 10^5^ to 10^6^ cells from ART-treated RMs).

### In situ hybridization

Tissues were frozen in isopentane, cooled in liquid nitrogen and then cryostat sectioned at 4-μm intervals. Viral replication in tissues was assessed by in situ hybridization using a ^35^S-labeled RNA probe derived from the transcription vector Bluescript in which a fragment of the SIVmac142 clone spanning the nef coding region was inserted.^57,83^ The antisense probe used to detect SIV RNA was generated from the T7 promoter by in vitro transcription of plasmid template with 50 units of T7 RNA polymerase in the presence of 50 µCi of [^35^S]UTP and [^35^S]ATP. Specific activity ranged between 1 × 10^8^ and 5 × 10^8^ cpm/µg of input DNA.

### In vitro SIV production

From SIV-infected RMs, TCM, TEM and TFH sorted cells (10^5^ cells) were activated using plate-bound anti-CD3 (0.5 µg/well; Lifetechnologies) and anti-CD28 mAbs (1 µg/well; Biolegend). Cells were cultured in media (RPMI 1640, 10% fetal bovine serum, 1 mM of sodium pyruvate and 1% of penicilline–streptomycin–glutamine at 50 mg/ml; Lifetechnologies) at 37 °C with 5% of CO_2_. At day 4 post activation, supernatants were harvested to measure viral production or to initiate viral infection using the CEM×174 cell line. At day 4 after this new round of infection, viral DNA was quantified in CEM×174 cells as described above.

### Statistical analysis

Statistics were performed using the GraphPad Prism 8 software. The nonparametric Mann–Whitney test and the paired *t* test were employed for comparison as indicated in the figures. *P* value < 0.05 indicates a significant difference.

## Supplementary information

Supplementary Tables

Supplementary Information
