# Supplementary material for: Despite early antiretroviral therapy effector memory and follicular helper CD4 T cells are major reservoirs in visceral lymphoid tissues of SIV-infected macaques
Source: Mucosal Immunol. 2019 Nov 13;13(1):149–60. doi: 10.1038/s41385-019-0221-x (PMC6914669; doi:10.1038/s41385-019-0221-x)
Supplement: Supplementary file 1 — Supplementary Tables [file 41385_2019_221_MOESM1_ESM.pdf]

Cluster of differentiation	Fluorochrome	Clone	Manufacturer
CD279	FITC	EH12.2H7	Biolegend
CD185	PE	87.1	eBioscience
CD45RA	EDC	2H4LDH11LDB9	Beckman Coulter
CD197	PE Cy7	3D12	BD Biosciences
CD3	Alexa 700	SP34-2	BD Biosciences
CD8	APC Cy7	SK1	BD Biosciences
CD4	V500	L200	BD Biosciences
CD45RA	PE Cy7	5H9	BD Biosciences
CD197	Percp Cy5.5	G043H7	Biolegend
CD20	APC-H7	2H7	BD Biosciences
CD32	BV421	FLI8.26	BD Biosciences

Table S1 : Antibody used.

	Primers and probes	Nucleotides sequences 5'-3'
SIV	SIVmac-F	GCA GAG GAG GAA ATT ACC CAG TAC
	SIVmac-R	CAA TTT TA CCC AGG CAT TTA ATG TT
	SIVmac-Probe	6FAM TGT CCA CCT GCC ATT AAG CCC GA TAMRA
	EarlySIV-For	AAG CTA GTG TGT GTT CCC ATC T
	EarlySIV-Rev	CTT CGG TTT CCC AAA GCA GAA
	SIV DNA-Preco	CAG AGG CTC TCT GCG ACC CTA C
	SIV DNA-K3	GAC TGA ATA CAG AGC GAA ATG C
	SIV DNA-For	TCC CTA GGA GGA TTA GAC AAG G
	SIV DNA-Rev	CTC TCT TCA GCT GGG TTT CTC
	SIV DNA-Probe	56FAM AGC TCA CTC ZEN TCT TGT GAG GGA CAG A 3IABkFQ
	ADN18S-For	CCT CCA ATG GAT CCT CGT TA
	ADN18S-Rev	AAA CGG CTA CCA CAT CCA AG
HIV	EarlyHIV-For	AGC CTG GGA GCT CTC TGG CTA
	EarlyHIV-Rev	CCA GAG TCA CAC AAC AGA CGG
	ULF1	ATG CCA CGT AAG CGA AAC TCT GGG TCT CTC TDG TTA GAC
	UR1	CCA TCT CTC TCC TTC TAG C
	LAMBDA T	ATG CCA CGT AAG CGA AAC T
	UR2	CTG AGG GAT CTC TAG TTA CC
	UHIV Taqman	6FAM CAC TCA AGG CAA GCT TTA TTG AGG C TAMRA
	ADN18S-For	CCT CCA ATG GAT CCT CGT TA
	ADN18S-Rev	AAA CGG CTA CCA CAT CCA AG

Table S2: Primers and probes used.
