# Supplementary material for: Despite early antiretroviral therapy effector memory and follicular helper CD4 T cells are major reservoirs in visceral lymphoid tissues of SIV-infected macaques
Source: Mucosal Immunol. 2019 Nov 13;13(1):149–60. doi: 10.1038/s41385-019-0221-x (PMC6914669; doi:10.1038/s41385-019-0221-x)
Supplement: Supplementary file 2 — Supplementary Information [file 41385_2019_221_MOESM2_ESM.pdf]

SUPPLEMENTAL INFORMATION

Supplemental figure 1. Lymphoid tissues included in this study: Representative pictures of peripheral A) axillary/inguinal LNs, B) spleen and C) mesenteric LNs along the mesentery and the colon (large intestine).

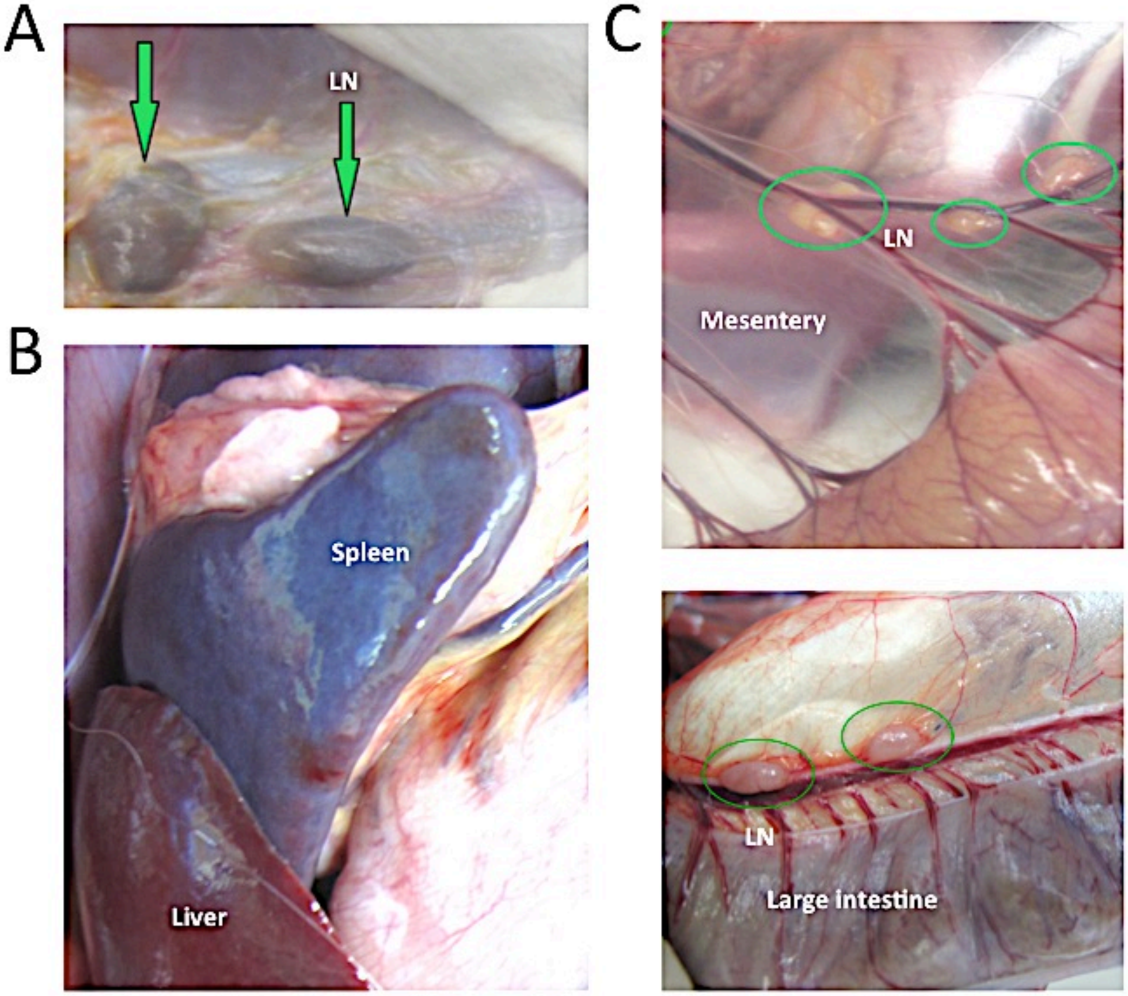

Supplemental figure 2. Cell sorting strategy: Representative dot plots depicting the gating strategy used to isolate CD4 T cell subsets. $CD3^+$ T cells were separated in $CD3^+CD20^-$ and then in $CD3^+CD4^+$ *versus* $CD3^+CD4^-$ T cells. Gating on $CD3^+CD4^+$ T and based on the expression of CXCR5 and PD-1 we isolated TFH cells ($CXCR5^+PD\text{-}1^{bright}$). Non-TFH cells were then separated in naive (TN, $CD45RA^+CCR7^+$), central memory (TCM, $CD45RA^-CCR7^+$), effector memory (TEM, $CD45RA^-CCR7^-$) and terminally differentiated (TTD, $CD45RA^+CCR7^-$) subpopulations.

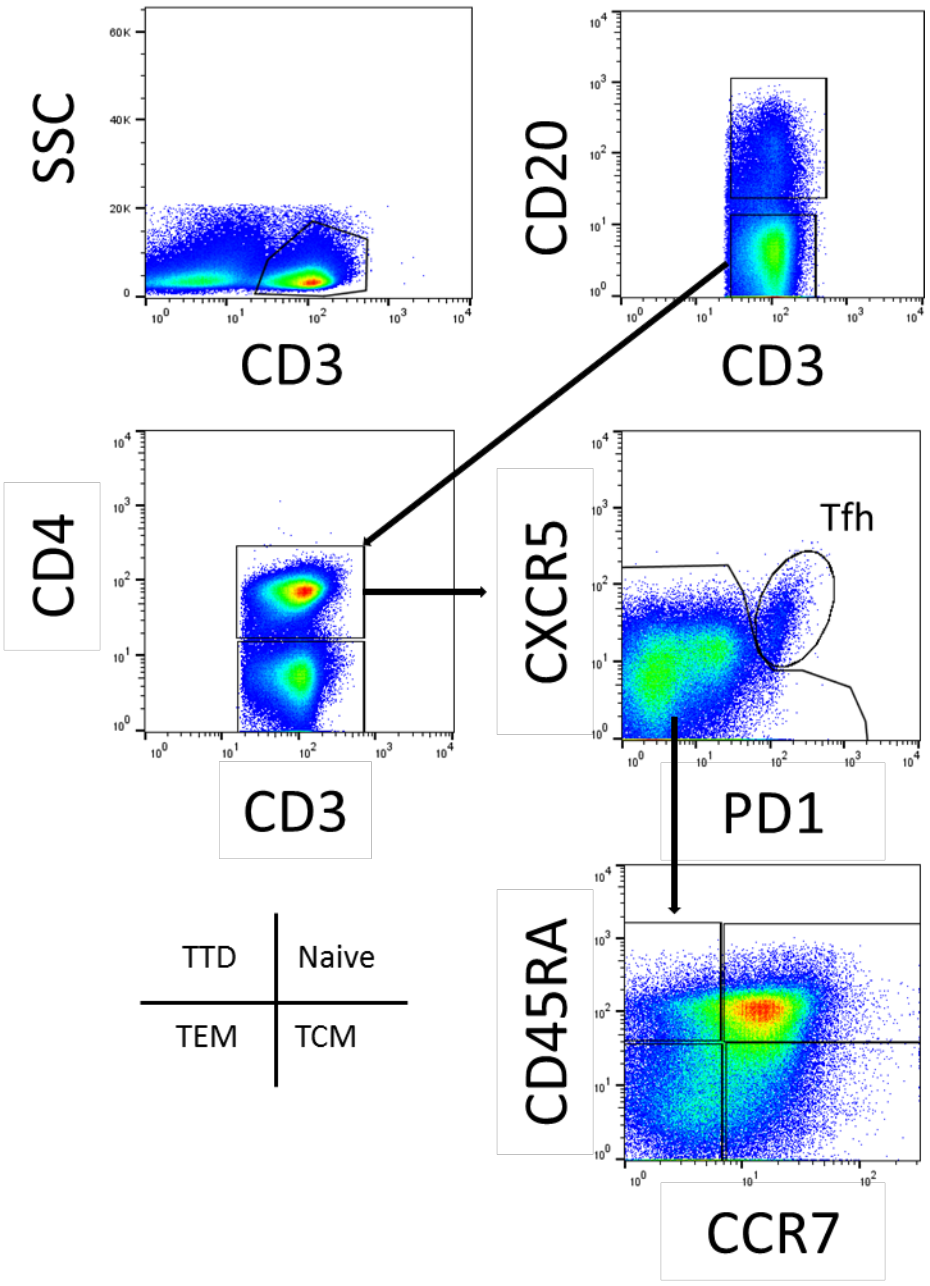

Supplemental figure 3. Contents of reverse transcripts (R) and of SIV DNA (D) in Naïve, TCM, TEM, TFH and TTD populations from the spleen, mesenteric LNs (Mes LN) and peripheral LNs (Ax-Ing LN) from RMs at the **A**) acute (days 11-14) and **B**) steady state phases (days 18-30) of infection. Reverse transcripts and DNA are expressed as copies per 10^4 cells. Each symbol represents one individual. Statistical analyses were performed using paired t-test to compare vDNA (D, p value in red) and early reverse transcripts (R, p value in blue) in the different subsets as well between E versus D (p value in black). * indicates $p<0.05$ and ** for $p<0.01$.

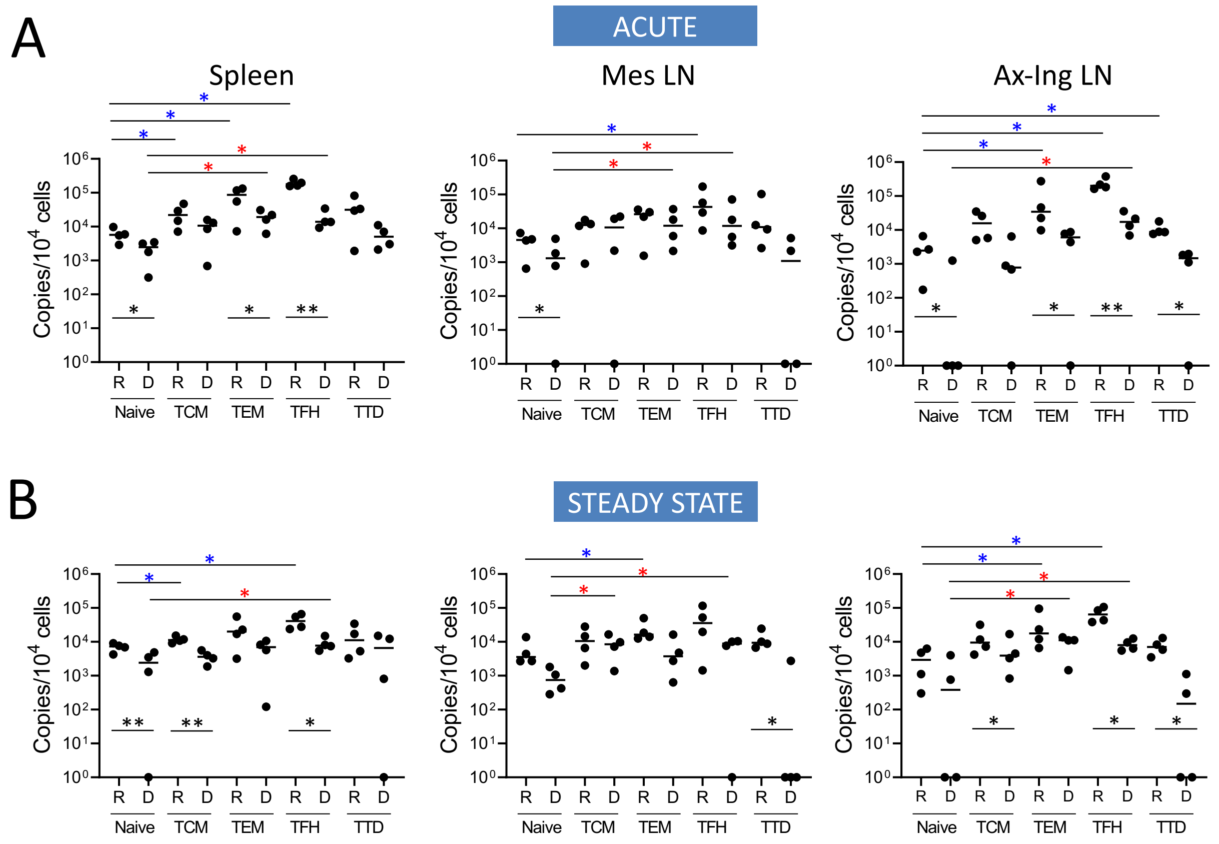

Supplemental figure 4. Contents of SIV RNA in Naïve, TCM, TEM, TFH and TTD populations from the spleen, mesenteric LNs (Mes LNs) and peripheral LNs (Ax-Ing LNs) from RMs at the **A**) acute (days 11-14) and **B**) steady state phases (days 18-30) of infection. SIV RNA is expressed as copies per 10^4 cells. Each symbol represents one individual. Statistical analyses were performed using Mann Whitney test. * indicates $p<0.05$.

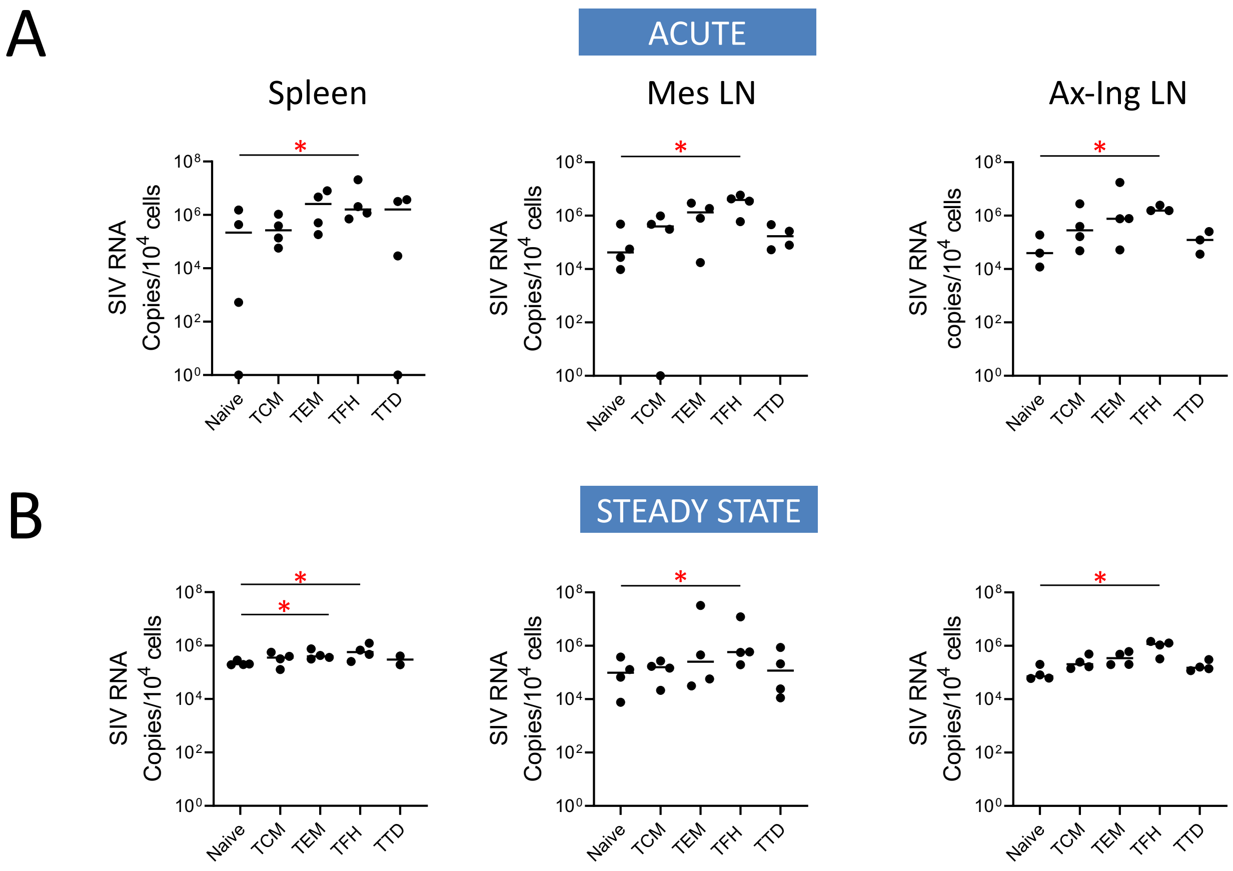

Supplemental figure 5. Productive SIV infection: SIV RNA$^+$ cells were detected by in situ hybridization in **A**) mesenteric LN and **B**) the spleen of a SIV-infected RM (#PB041) sacrificed at day 11 post-infection. Infected cells are mainly localized in the follicles of lymphoid tissues.

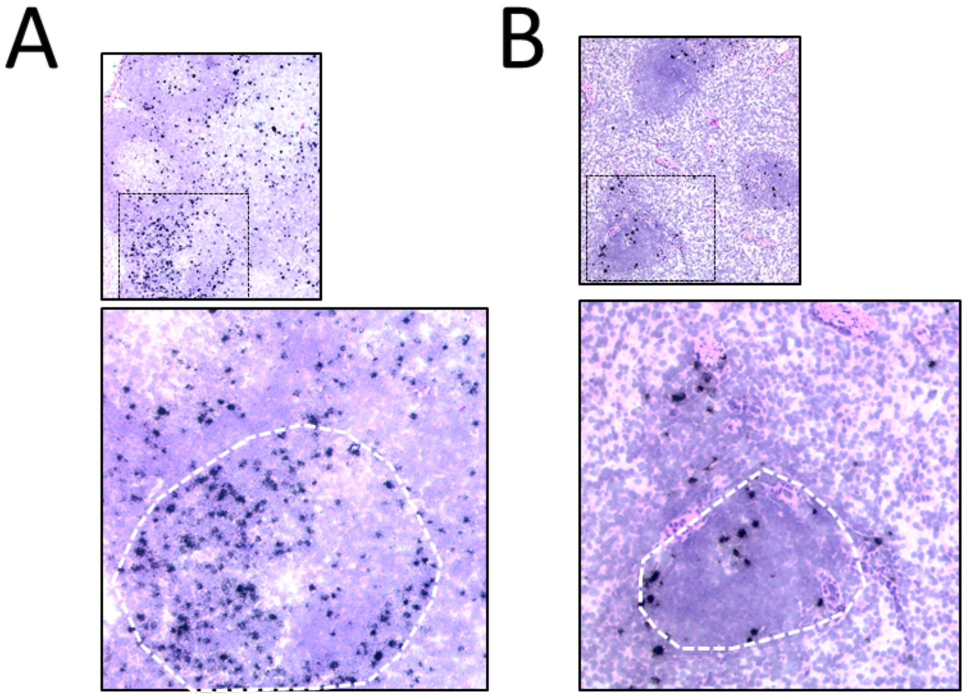

Supplemental figure 6. Productive infected CD4 T cell subpopulations. A) Levels of viral DNA were quantified by RT-qPCR in sorted TCM, TEM and TFH cells from mesenteric LNs of SIV-infected RMs (n=3). **B)** Cells were activated with plate-bound anti-CD3 and anti-CD28 antibodies. After 4 days, viral production was evaluated by RT-qPCR in the supernatants. **C)** To evaluate that virions produced are infectious, supernatants were co-cultured with CEMx174 cells. DNA results are expressed as copies per 10^4 cells at day 4 post-infection. A paired t-test was performed, * indicates $p<0.05$.

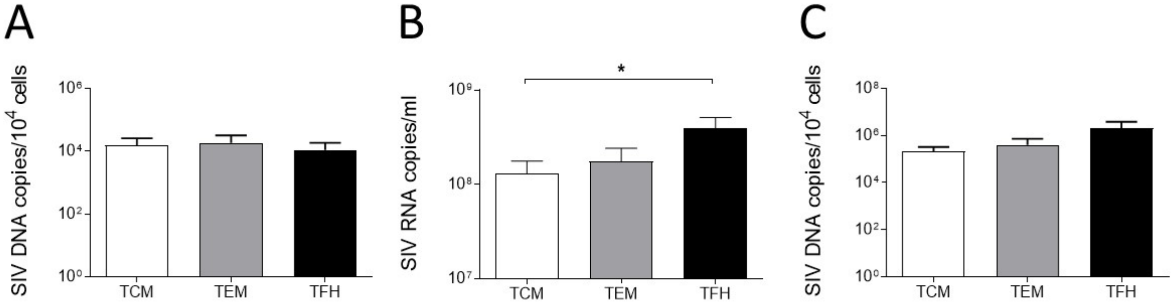

Supplemental figure 7. CD4 T cell numbers in lymphoid tissues. Numbers of CD4 and of TCM, TEM and TFH subsets are shown for spleen, mesenteric LNs (MES LNs) and peripheral LNs (AX+ING LNs). Each symbol represents one individual from either non-infected or SIV-infected RMs during the acute (days 11-14) and steady state (days 18-30) phase of the infection. Cell numbers were calculated considering total cell numbers obtained for each tissue and the percentage of each population determined by flow cytometry. A Mann Whitney test was performed, * indicates $p<0.05$; **, $p<0.01$.

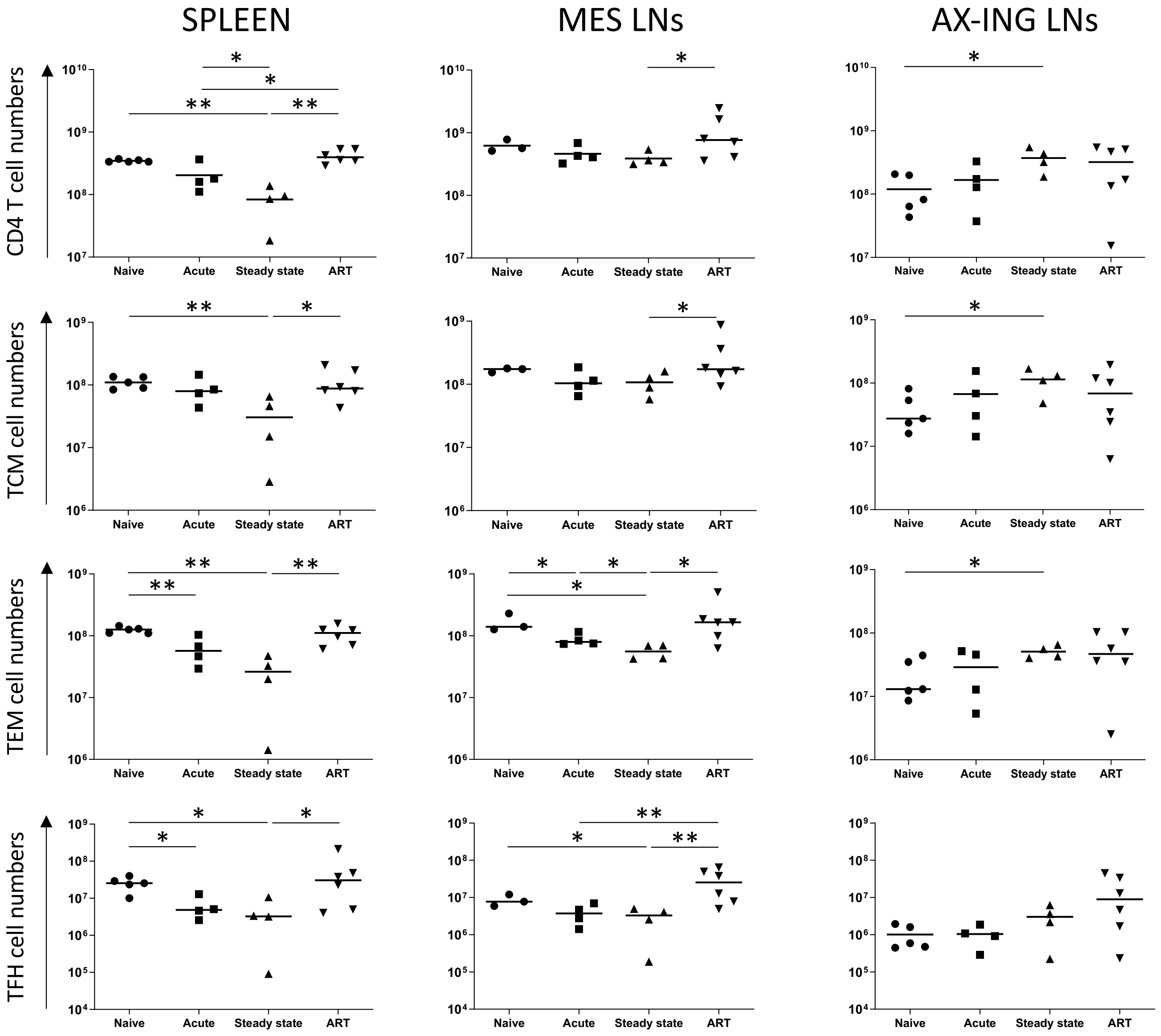

Supplemental figure 8. Detection of SIV DNA and R-U5 in RMs treated with ARV at week 6 post-infection. (A-C) RMs (n=12) were treated with ARV at week 6 post-infection. ARV consists in a cocktail of TFV, FTC, DTG administrated daily subcutaneously, and of DRV administrated by oral route, twice daily. CD4 T cells were isolated from (A) peripheral blood mononuclear cells (PBMC) and (B) lymph nodes (LNs) of ART-treated RMs at weeks 14 and 32 post-infection. Viral DNA and SIV R-U5 were quantified by qPCR. Each dot represents one individual. Due to the low amount of blood and LN materials, only 11 blood RMs were tested at week 14 and 9 LN RMs at week 32. A Mann Whitney test was performed, * indicates $p<0.05$; **, $p<0.01$; ***, $p<0.001$ and ****, $p<0.0001$. **C)** Furthermore, RMs were sacrificed and CD4 T cells were sorted from PBMC, LNs and spleen. Results are expressed as copies per 10^4 cells. A paired t-test was performed, **, $p<0.01$.

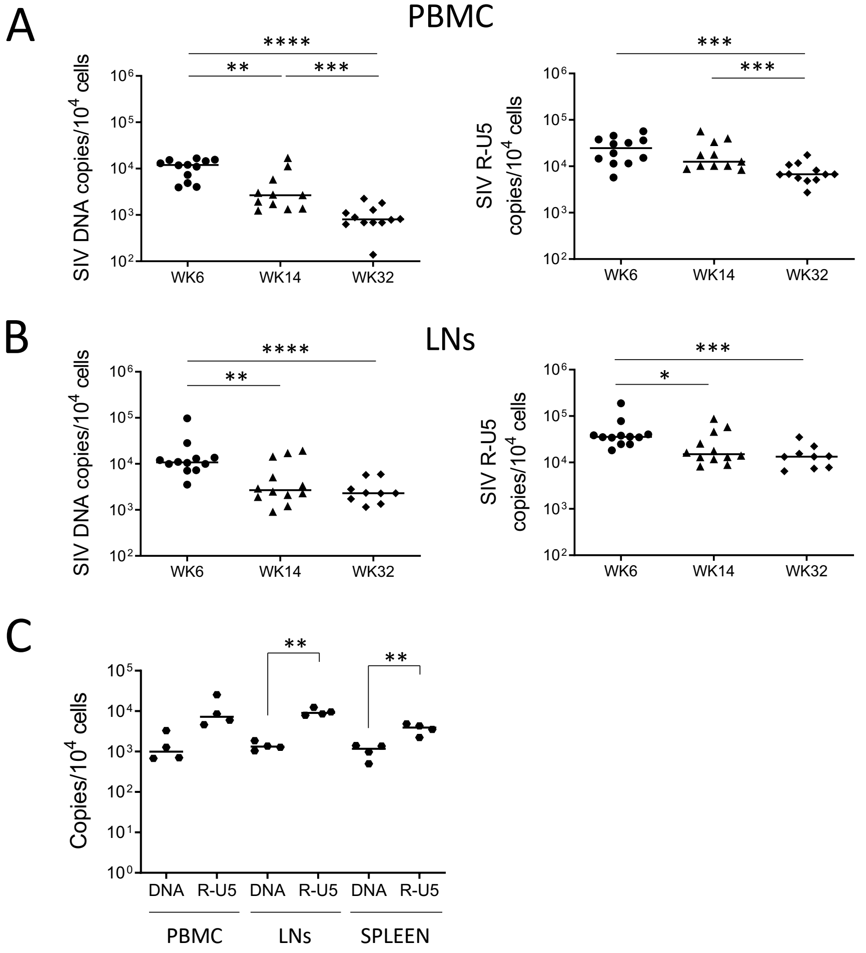

Supplemental figure 9. CD32 populations. A) Representative dot plots depicting the gating strategy used to analyze CD32 expression in CD4 T cell populations from mesenteric LNs of ARV-treated RMs. $CD3^+$ cells were separated in $CD3^+CD20^+$ *versus* $CD3^+CD20^-$ cells. Gating on $CD3^+CD20^+$ population, $CD32^+$ was analyzed on $CD4^+$ cells. Furthermore, gating on $CD3^+CD20^-$cell subset, the cells were then separated in TFH ($CXCR5^+PD\text{-}1^{bright}$) and Non-TFH cells. These latter were then separated in naive (TN, $CD45RA^+CCR7^+$), central memory (TCM, $CD45RA^-CCR7^+$), effector memory (TEM, $CD45RA^-CCR7^-$) and terminally differentiated (TTD, $CD45RA^+CCR7^-$) subpopulations. Similar to the $CD3^+CD20^+$ population, we identified CD32 expression in each subset. **B)** Percentages of CD32 expression and viral DNA (copies/10^4) in each T cell subsets from two ARV-treated RMs.

A

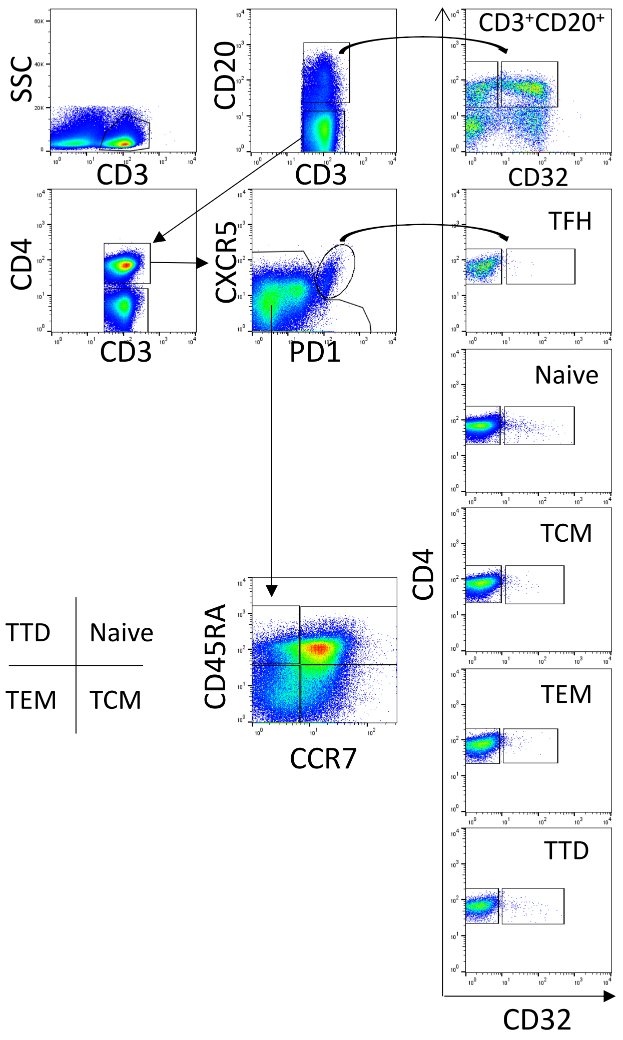

B

	#1		#2	
	$CD32^+$ %	DNA^+ 10^4 cells	$CD32^+$ %	DNA^+ 10^4 cells
$CD3^+CD20^+$	28.00	0.00	11.00	0.00
TFH	0.62	0.00	0.20	302.91
Naive	0.40	0.00	0.18	0.00
TCM	0.22	0.00	0.16	57.65
TEM	0.63	34.14	0.31	151.82
TTD	1.03	0.00	0.29	0.00
